# Integrated analysis of diastolic, systolic and pulmonary vascular function using MRI guided catheterization

**DOI:** 10.1186/1532-429X-11-S1-O9

**Published:** 2009-01-28

**Authors:** Boris Schmitt, Paul Steendjik, Karsten Lunze, Stanislav Ovrouski, Jan Falkenberg, Pedram Rahmanzadeh, Nizar Maarouf, Peter Ewert, Felix Berger, Titus Kuehne

**Affiliations:** 1 Unit of Cardiovascular Imaging – Congenital Heart Diseases, Deutsches Herzzentrum Berlin, Berlin, Germany; 2 Cardiology, Medical University Leiden, The Netherlands Leiden, Netherlands

**Keywords:** Vascular Resistance, Dobutamine, Diastolic Dysfunction, Pulmonary Vascular Resistance, Vascular Function

## Indroduction and purpose

An integrated approach for assessing ventricular pump function, diastolic compliance (EDPVR), myocontractility and pulmonary vascular resistance would be of clinical interest. In addition to pump function, MRI guided catheterization was demonstrated to accurately measure myocontractility and vascular resistance. We now extended this method for acquisition of the EDPVR. Subsequently, this approach was applied in patients with Fontan hemodynamic in which abnormalities in pulmonary vascular, myocontractile and diastolic properties are debated.

## Methods

The EDPVR was determined by synchronizing invasive ventricular pressures with cine and real-time MRI derived ventricular volumes and pulmonary/aortic blood flow measurements.

### Validation part

In 7 pigs the MRI and conductance-catheter method (gold standard) were compared for measuring the EDPVR at rest and during dobutamine.

### Clinical part

Parameters of global function, myocontractility (ESPVR), vascular resistance and EDPVR were measured with MRI at rest and under dobutamine in 14 patients with Fontan circulation.

## Results

Bland-Altman test showed agreement between the conductance-catheter and MRI method. In the pigs, there was in both ventricles during dobutamine a right/bottom shift of the EDPVR, the stiffness co efficient decreased slightly (p < 0.051). In the patients during dobutamine we noted failure to increase stroke volumes despite increased contractility and evidenced diastolic dysfunction. Active relaxation was inconspicuous but the EDPVR shifted towards the left/top, the stiffness constant remained unchanged. Pulmonary resistance decreased slightly (p = 0.058) and thus showed adequate response to augmented cardiac outputs.

## Conclusion

This novel MRI method provides differential information about diastolic, systolic and vascular function. The method evidenced that in Fontan patients diastolic dysfunction is an important pathophysiologic cause of heart failure.

